# Unravelling the Genetic Basis of Moisture Deficit Stress Tolerance in Wheat for Seedling Vigour-Related Traits and Root Traits Using Genome-Wide Association Study

**DOI:** 10.3390/genes14101902

**Published:** 2023-09-30

**Authors:** S Ramappa, Monika A. Joshi, Hari Krishna, Vijay Dunna, Neelu Jain, Rohini Sreevathsa, Narayana Bhat Devate

**Affiliations:** 1 Division of Seed Science and Technology, ICAR-Indian Agricultural Research Institute, New Delhi 110012, India; 2 Division of Genetics, ICAR-Indian Agricultural Research Institute, New Delhi 110012, India; 3 Division of Molecular Biology and Biotechnology, ICAR-Indian Agricultural Research Institute, New Delhi 110012, India

**Keywords:** candidate genes, genome-wide association study (GWAS), moisture deficit stress, seedling vigour traits, SNPs, wheat

## Abstract

A key abiotic stress that negatively affects seed germination, plant development, and crop yield is moisture deficit stress. Achieving higher vigour and uniform germination under stress conditions is essential for crop establishment and productivity and to enhance the yield. Hence, revealing wheat’s capacity to withstand moisture deficit stress during seed germination and early growth stages is fundamental in improving its overall performance. However, the genetic regulation of moisture deficit stress tolerance during the seed germination phase remains largely unexplored. In this study, a total of 193 wheat genotypes were subjected to simulated moisture deficit stress using PEG-6000 (−0.4 MPa) during the seed germination stage. The induced moisture deficit stress significantly reduced various seedling-vigour-related traits. The genetic regions linked to these traits were found using a genome-wide association study (GWAS). The analysis identified 235 MTAs with a significance −log10(p) value of >4. After applying the Bonferroni correction, the study identified 47 unique single nucleotide polymorphisms (SNPs) that are linked to candidate genes important for the trait of interest. The current study emphasises the effectiveness of genome-wide association studies (GWAS) in identifying promising candidate genes, improving wheat seedling vigour and root traits, and offering essential information for the development of wheat cultivars tolerant to moisture deficit stress.

## 1. Introduction

Wheat (*Triticum aestivum* L.) is a cereal crop of great significance that is cultivated in almost every part of the world. It is the staple food for around 2.5 billion people in nearly 90 countries worldwide [1]. To fulfil the growing demand for wheat consumption driven by the rising world population, wheat production has to be increased [2]. The abiotic stress condition is a common issue around the world, with moisture deficit stress being the most significant factor that hinders crop growth. This type of stress is becoming increasingly prevalent, especially in arid and semi-arid regions [3]. Plant performance and yield are clearly impacted by moisture deficit stress, with the germination and seedling growth stages of the majority of crops being the most prone to such conditions [4,5]. Early embryonic exposure to moisture deficit stress can cause seed germination to be delayed and germination rates to decline [4,6]. 

High productivity is ensured by superior quality seed, which is considered an essential aspect in boosting agricultural production [7]. One of the most significant variables that influences seed quality is seedling vigour, which has a direct impact on crop productivity by determining the genetic as well as yield potential of the seed while ensuring uniformity in seed germination, seedling growth, seedling establishment in the field, and withstanding sub-optimal environmental conditions [8]. Seedling vigour, determined by various physiological growth parameters, is a complex trait influenced by multiple quantitative trait loci (QTLs). In recent decades, QTL analysis has become a potent tool for studying the inheritance of such intricate traits. Through QTL analysis, researchers have successfully identified genetic regions associated with seedling vigour, revealing their correlation with a wide range of physiological traits such as germination rate, shoot length, seedling dry weight, mesocotyl length, coleoptile length, germination potential, germination index, root length, and root system architecture [9,10,11,12]. Root traits also play a crucial role in absorbing water during moisture deficit stress, taking up soil nutrients, and tolerating environmental stress [13]. The spatial organisation of the root system architecture is determined by basic traits including root length, root length density, root diameter, surface area, and volume. These traits have a strong relationship with how much water and nutrients are absorbed [14,15,16]. Since thicker roots with larger xylem vessels are more efficient at drawing water and nutrients from deep soil layers under rain-fed conditions, drought tolerance is directly correlated with root diameter [15]. The efficiency of water and nutrient absorption could be improved by increasing the surface area and volume of the roots, fine roots, and number of root tips, which are essential components of root systems [13,17]. Deep rooting, a crucial root characteristic that allows plants to access water present in the deeper soil layers, can improve the crop yield [18]. To enhance tolerance to moisture deficit stress, alleles for deeper root systems can be identified and introduced into drought-vulnerable shallow-rooted cultivars [19,20,21]. Therefore, by investigating root system architecture (RSA), we can find proxy characteristics that increase tolerance to diverse soil types and address moisture and nutrient stress situations [22]. Root system architecture (RSA) traits are influenced by environmental conditions and are determined by polygenes with cumulative effects [15]. However, the optimisation of RSA traits has been neglected due to the challenges in accurately assessing them in field conditions [15,16,23]. Nevertheless, researchers are studying the root architecture of seedlings as it is associated with the root structure of adult plants, aiming to apply this knowledge to later stages of growth [24,25]. The main aim of breeding is consistently to enhance yield. Several studies have explored quantitative traits linked to moisture deficit stress tolerance in wheat during its productive phase. For instance, QTL investigations in wheat have delved into the genetic factors governing yield and related traits like kernel weight, grain count per spike, grain yield, grain size, spike length, and spike shape [26,27,28]. The genetic basis underlying complex traits was deciphered using an effective method, GWAS. 

GWAS is a highly effective approach for detecting genes/QTLs through linkage disequilibrium (LD). Wheat, a self-pollinated crop, has a higher level of LD across its genome, resulting in high resolution and power of association [29]. Utilising genome-wide-dense markers, GWAS has been extensively used in several crops to identify candidate genes for several complex traits [30,31,32,33,34,35,36]. The benefits of GWAS include being capable of more effectively, and at less cost than biparental QTL mapping, uncovering MTAs/QTLs with high resolution, utilising diverse genotypes [37]. One of the most effective ways to find robust QTLs that have an impact in both normal and stressful conditions is via GWAS [38,39,40]. GWAS has, thus, developed into a potent and extensively used technique for investigating complex traits [41]. It involves genotyping a large collection of genotypes with many SNPs spread throughout the genome and examining the relationships between these markers and agronomic characteristics [42]. However, only a few studies have utilised the GWAS methodology to examine variables associated with seedling-vigour-related traits in wheat genotypes under moisture deficit stress. In order to uncover the underlying genetic factors governing variations in these traits, the current work aims to investigate the genetic variation of and traits associated with seedling vigour and root traits under artificially induced moisture deficit stress conditions. This investigation contributes to the understanding of the genetic regulation of naturally occurring variations in traits related to moisture deficit stress tolerance, providing a foundation for subsequent genetic investigations. Here, we discovered new SNP markers linked to the natural variation in variables related to seedling vigour and root traits.

## 2. Results

### 2.1. Phenotypic Evaluation

**Figure 1:**
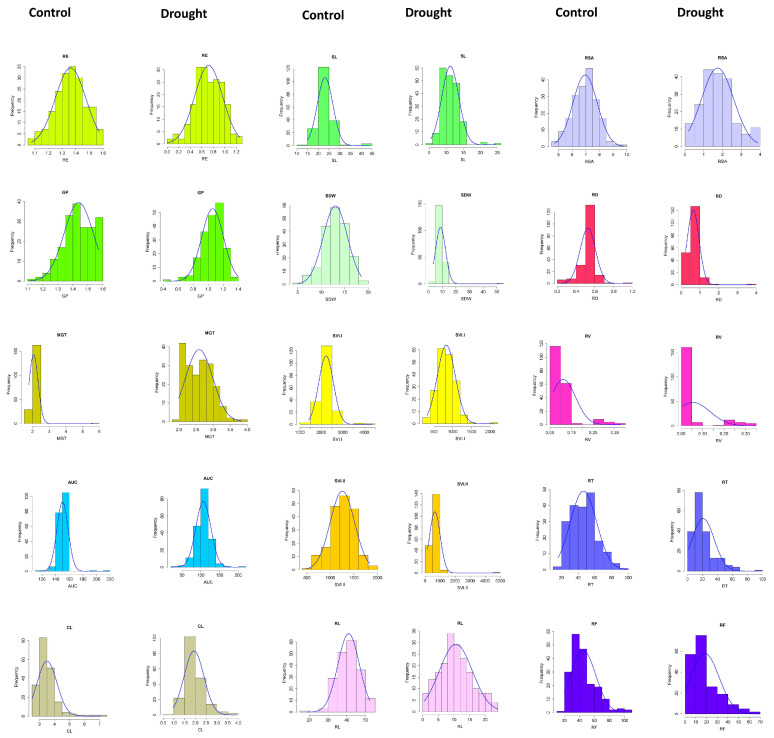
Histogram showing the frequency distribution of all the studied traits across the seasons for the control and moisture deficit stress conditions.

Due to the substantial diversity, significant differences were observed among various seedling vigour and root traits in the wheat association panel (Table 1). As shown in Table 1, an analysis of variance (represented as an MSS, or “mean sum of squares”) revealed a significant difference between the investigated traits at α values of 0.05 and 0.01. The differences were significant between genotypes as well as treatments and also the interaction effects showed significant variation across the seasons; only RE, RSA, and RD did not show any seasonal variation. Mean values (mean ± SE) of all the traits for the genotypes were as follows: RE (1.04 ± 0.01), GP (1.25 ± 0.01), MGT (2.33 ± 0.02), AUC (128.45 ± 0.85), CL (2.72 ± 0.04), SL (17.18 ± 0.20), SDW (10.93 ± 0.19), SVI.I (1525.99 ± 17.12), SVI.II (954.73 ± 17.51), RL (25.79 ± 0.27), RSA (4.34 ± 0.04), RD (0.58 ± 0.01), RV (0.08 ± 0.00), RT (33.66 ± 0.82), and RF (31.70 ± 0.82). We observed that the coefficient of variation (%CV) ranged from 7.36% to 75.41% for all the phenotypic traits (Table 1). The study found that the lowest CV was observed for seedling vigour traits, viz., GP (7.36%), followed by AUC (9.22%) and MGT (9.94%). On the other hand, the highest CV was observed for root traits, viz., RV (75.41%), followed by RF (35.83%) and RT (33.86%) (Table 1). Under control and moisture deficit conditions, significant differences were recorded for all the investigated traits and the genotype × season interaction effect was also observed. However, seasonal variation and the genotype × season interaction effect were absent in the control condition for the traits MGT and SVI.I (Supplementary Tables S2 and S3). The frequency distribution histogram with density curve for all the traits under the two conditions is given in Figure 1. In the control condition, the traits MGT, CL, AUC, RL, and RV displayed a skewed distribution, indicating an uneven distribution of data points around the central value. However, under the moisture deficit stress condition, the traits GP, SDW, SVI.II, RD, RV, RT, and RF also showed a skewed distribution, indicating a similar uneven pattern of data distribution in response to the stress (Figure 1).

### 2.2. Correlation Principal Component Analysis

**Figure 2:**
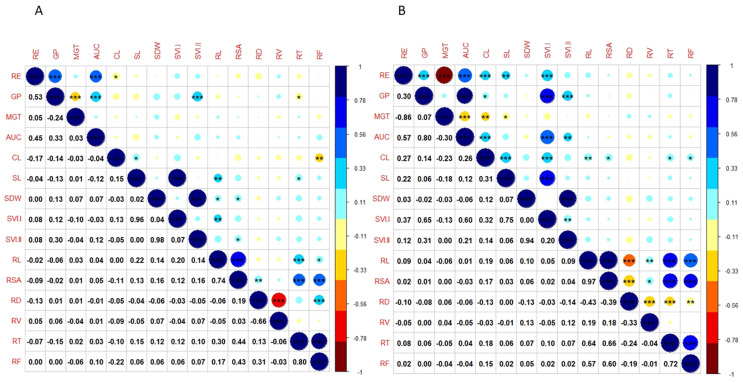
Correlations between seedling-vigour-related traits and root traits. (**A**): control; (**B**): PEG treatment; RE: radicle emergence (%); GP: germination percentage (%); MGT: mean germination time (d); AUC: area under the curve; CL: coleoptile length (cm); SL: seedling length (cm); SDW: seedling dry weight (mg); SVI.I: seedling vigour index-I; SVI.II: seedling vigour index-II, RL; total root length (cm); RSA: root surface area (cm^2^); RD: average root diameter mm; RV: root volume (cm^3^); RT: number of root tips; RF: number of root forks; *** 0.1% level of significance; ** 1% level of significance; * 5% level of significance.

A correlation analysis revealed the presence of relatedness among the studied traits (Figure 2). A highly significant positive correlation is observed among RE, GP, AUC, and RV, whereas traits like SL, SVI.I, RL, RSA, RT, and RF showed a positive correlation among them under the control condition. CL has a negative correlation with most of the studied traits except SL and SVI.I. The SVI.II is positively correlated with most of the root traits except RD under the control condition. Total root length (RL) is positively correlated with most of the seedling vigour traits, viz., MGT, AUC, SL, SDW, SVI.I, and SVI.II, but not with RE, GP, or CL (Figure 2A). Whereas in the case of the drought condition, positive correlation is observed among RE, GP, AUC, CL, SL, SDW, and SVI.I, but RE is negatively correlated with MGT, RD and RV. The root traits such as RL, RSA, and RT have a positive correlation with the seedling vigour traits except MGT (Figure 2B).

**Figure 3:**
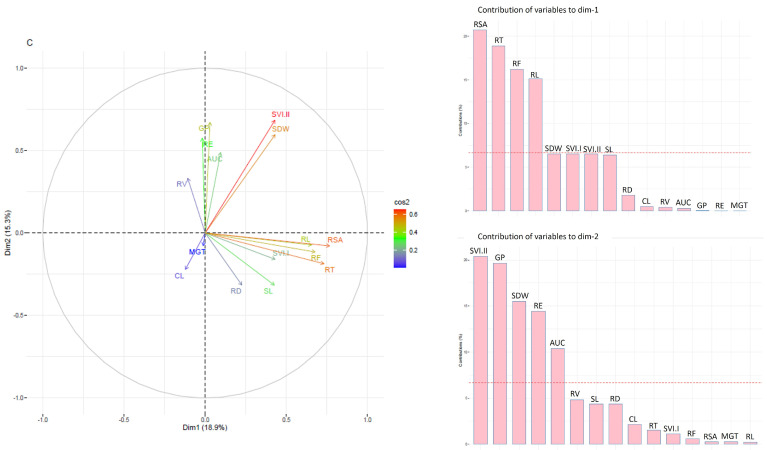
PCA biplot and contribution of studied traits to dimension 1 and dimension 2 in GWAS panel evaluated under control condition (**C**) across the two seasons.

**Figure 4:**
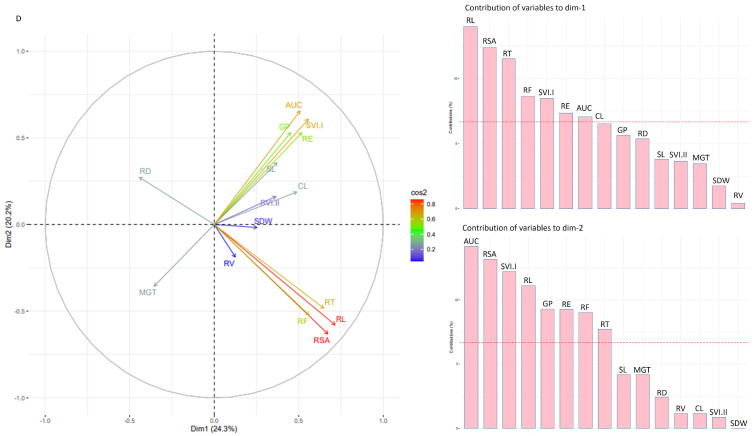
PCA biplot and contribution of studied traits to dimension 1 and dimension 2 in GWAS panel evaluated under moisture deficit condition (**D**) across the two seasons.

In the PCA analysis using phenotypic data under control conditions, the first principal component accounted for 18.9% of the total variation. The primary factors driving this component were the root traits: RSA, RT, RF, and RL (Figure 3). On the other hand, the second dimension explained 15.3% of the variation and was influenced by traits such as SVI.II, GP, SDW, RE, and AUC (Figure 3). The traits RE, GP, AUC, and RV were observed to cluster closely together with an acute angle, indicating a strong positive correlation among them. Similarly, RL, RSA, RT, and SVI.I showed a similar clustering pattern, suggesting a positive correlation among these traits as well. On the other hand, CL, which is separate from the positively correlated clusters, displays a negative correlation with most of the studied traits, except SL and SVI.I. Similarly, PCA analysis under the moisture deficit stress condition indicated that dimension 1 explained 24.3 and dimension 2 explained 20.2% of the variation. The first dimension of variability was influenced by root traits such as RL, RSA, RT, RF, SVI.I, RE, and AUC. On the other hand, the second dimension of variability was associated with traits like AUC, RSA, SVI.I, RL, GP, RE, RF, and RT (Figure 4). The traits RE, GP, AUC, CL, SL, SDW, and SVI.I are closely clustered together at an acute angle, suggesting a strong positive correlation among them. These traits show similar patterns of variation, indicating that when one of these traits increases, the others in this group are likely to increase as well.

Regarding the root traits RL, RSA, RT, and RF, they were also grouped, except for RD. This grouping indicates a consistent relationship among these root traits, while RD appears to behave differently and may have a distinct relationship with the other root traits (Figure 4).

### 2.3. Marker Trait Associations (MTAs)

For all the studied traits across the two seasons (seeds harvested from two *Rabi* seasons: 2020–21 and 2021–22) with different treatment conditions, viz., control and PEG treatment, 235 MTAs were identified with a significance −*log10(p)* value of >4. The highest number of SNPs was obtained for the trait SDW (31 MTAs) and the lowest for the RE (2 MTAs). MTAs were filtered with a Bonferroni correction value (−*log10(p)* > 5.45) to increase the stringency of selection, and 47 SNPs were obtained that were located on 18 different chromosomes (Table 2). Out of the 47 SNPs, 10 were identified across the seasons, which were linked with CL, SDW, and RD. Under control conditions, 10 SNPs linked with MGT, AUC, CL, and RD were obtained. Twenty-seven significant associations were obtained under the moisture deficit stress condition (PEG treatment) for traits, viz., AUC, SDW, SVI.I, RD, and RV (Table 2). The SNP AX-94471577, which is associated with MGT located on the 4A chromosome at 1.89 Mb, explained the highest phenotypic variation in PVE (99.87%). The AX-95194336 marker linked to AUC, located on chromosome 2B, at 9.62 Mb, was found to account for a significant 92.39% of the phenotypic variation explained (PVE). On the other hand, the MTA (AX-94446435) linked to RD on chromosome 3D had a PVE value of 0.0%, indicating the marker may not be used for the trait improvement. When subjected to moisture deficit stress conditions, the SNP marker AX-94742835 associated with SDW on chromosome 2D, at 90.24 Mb, demonstrated the highest phenotypic variation, with 47.13%. Conversely, under moisture deficit stress condition, the MTA (AX-95227434) linked to root diameter showed the lowest PVE, at 0.22% (Table 2).

**Figure 5:**
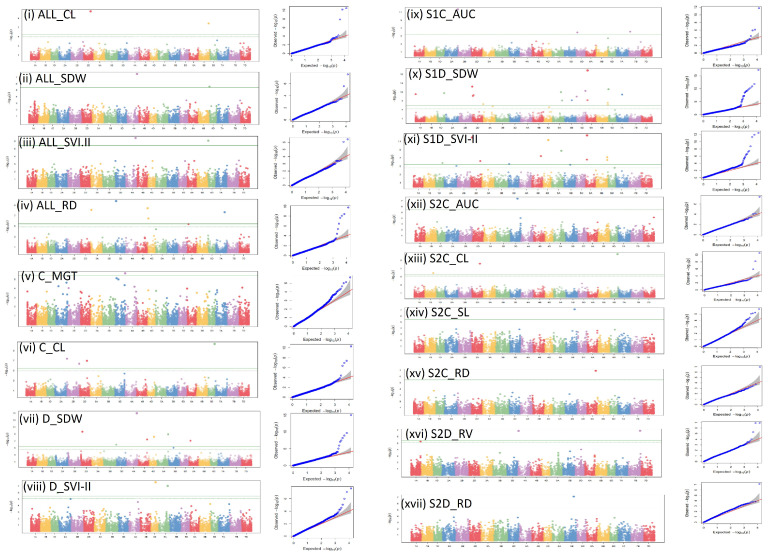
Manhattan and respective Q–Q plots of significant associations for seedling vigour and root traits under control, moisture deficit stress condition, and combined.

**Figure 6:**
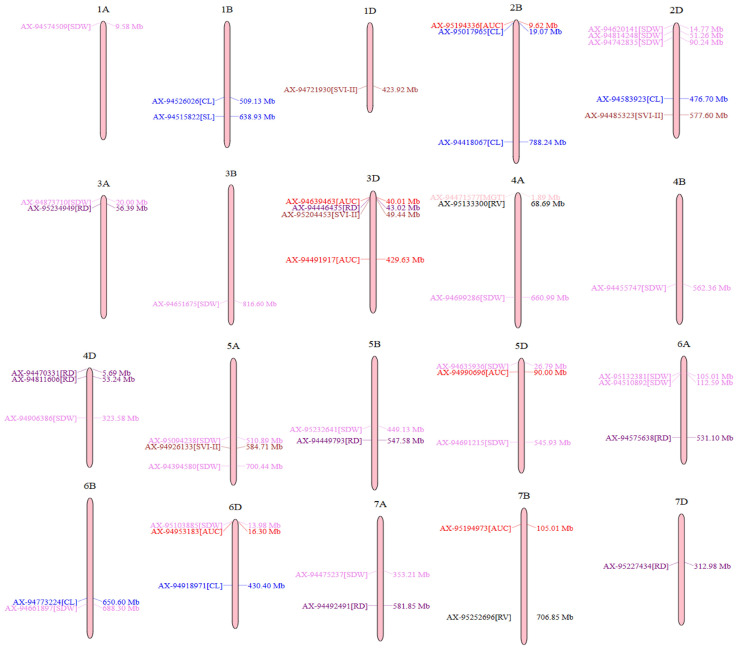
Distribution and position of identified MTAs (−log_10_(*p*) above 5.45) at their respective chromosome.

Visual depictions of significant SNPs identified across various seasons and treatments for the examined traits using Manhattan plots and Q–Q plots are presented in Figure 5. Additionally, the distribution and location of the identified significant MTAs (-*log10*(*p*) > 5.45) are displayed on their respective chromosomes (Figure 6). The details of the MTAs above Bonferroni correction with their position in the genome are noted down (Table 2). All the obtained SNPs were unique and linked to seven distinct traits: MGT, AUC, CL, SDW, SVI.I, RD, and RV. Chromosome 2D has the highest number of significant SNPs linked to traits. These SNPs are AX-94620141, AX-94814248, AX-94742835, AX-94583923, and AX-94485323. The least number of SNPs have been identified on chromosomes 1A (AX-94574509), 1D (AX-94721930), 4B (AX-94455747), and 7D (AX-95227434) (Table 2; Figure 6).

### 2.4. In Silico Analysis

The location of SNPs in the gene-rich region of the genome was identified by comparative analysis of important markers using BLAST against the Triticum aestivum reference genome from the IWGSC. Nearly all SNPs were found close to a transcript that encoded either a protein or transcription factor. SNPs were located near the genes coding for proteins like Peptidase S10, serine carboxypeptidase, UDP-glucuronosyl/UDP-glucosyltransferase, Alpha/Beta hydrolase fold, RmlC-like cupin domain superfamily, P-type ATPase, subfamily IIB, EF-hand domain, MFS transporter superfamily, etc. (Supplementary Table S4).

The SNP markers like AX-94583923, AX-95103885, AX-94811606, and AX-94394580 were linked to the genes governing traits like coleoptile growth, germination, seedling development, and seedling vigour, and are helpful in the early seedling vigour of the wheat. Similarly, SNPs such as AX-95017965, AX-94510892, and AX-95204453 are associated with CL, SDW, and SVI.I, while AX-95227434 and AX-94449793 are associated with RD. However, it is not known which genes and proteins they code for. Only one SNP marker associated with MGT, AX-94471577, was identified to be present near the candidate regions coding for WD40-repeat-containing domain superfamily, Protein DCL-like UDP-glucuronosyl/UDP-glucosyltransferase, and Aspartic peptidase domain superfamily. The coding regions were identified through in silico analysis. UDP-glucuronosyl/UDP-glucosyltransferase confers drought tolerance in spring wheat and plays an important role in root and stem development of abiotic and biotic stresses [43,44]. For AUC, AX-95194336, AX-95194973, and AX-94990696, SNPs were reported under the control, whereas AX-94953183 and AX-94639463 were reported in the moisture deficit stress condition in the first year (2020–21).

### 2.5. Multivariate Analysis of Wheat Genotypes Using MGIDI for Seedling Vigour and Root Traits under Both Control and Moisture Deficit Conditions

**Figure 7:**
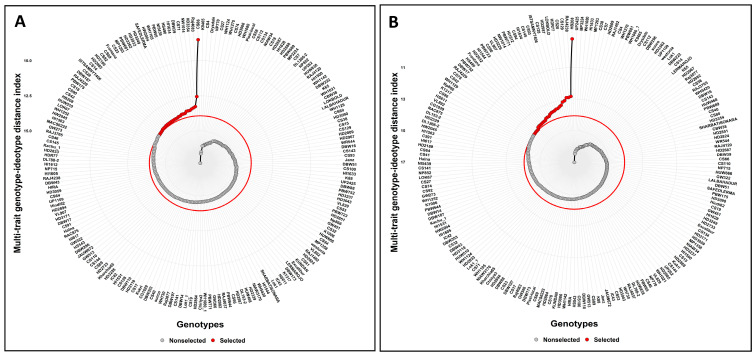
(**A**): Control; (**B**): moisture deficit condition. Arrangement of genotypes in ascending order according to the MGIDI index. Genotypes chosen using this index are highlighted in red. The central circle, in red, denotes the threshold point determined by the selection pressure.

The multi-trait genotype–ideotype distance index (MGIDI) was devised to assist in the selection of superior genotypes. This index considers both seedling vigour and root traits, allowing for the selection of the best genotypes under both control and moisture deficit stress conditions. The genotypes are displayed in ascending order using the MGIDI index. The genotypes chosen using this index are marked in red. The central circle, in red, denotes the threshold point determined by the selection pressure (SI = 15%). A total of 58 superior genotypes were selected under both the control and moisture deficit conditions, with 29 genotypes in each condition (Figure 7; Table 3). 

## 3. Discussion

In the current study, the genotypes used exhibited a wide range of phenotypic variation at the early developmental stage in response to moisture-deficit-induced stress. The population used is known for its high diversity and has been used in GWAS to detect QTL for quality and developmental traits such as grain micronutrients. Moisture deficit stress, induced by PEG, had a significant impact on most traits, likely due to decreased water uptake and delayed or reduced germination [45,46]. High germination rates and seedling vigour in wheat are crucial for stand development in the early stages under induced moisture deficit stress conditions. 

According to the results of the current study, drought reduces seedling- and root-related development, which has an adverse effect on seedling performance. It has been observed that the traits investigated under the stress conditions of induced moisture deficit have a wide range of genetic variability [47]. In cereals, strong early seedling vigour and well-developed root systems have been positively correlated with plant growth performance, both of which could improve stress tolerance [48,49]. When it comes to water access and uptake in the context of drought tolerance, root traits are very important. Thus, root traits support farm output maintenance in conditions with limited water availability. 

The presence of genetic diversity is an essential characteristic for the successful establishment and early vigour of seedlings, particularly in challenging environments with limited water and resources. The enhancement of root traits during the critical stages of seed germination and seedling development has a significant influence on the emergence of crops and the establishment of seedlings. This, in turn, leads to the development of a healthy plant population, which is a crucial factor for achieving high yields in both favourable and stressful conditions. Thus, emphasising these traits could prove to be an effective approach to quickly assess a large number of genotypes in a drought-like scenario. It could potentially be possible to uncover genotypes that perform better under moisture deficit stress conditions. In order to develop drought-tolerant cultivars through marker-assisted backcrossing, it is necessary to discover markers associated with the target traits. In this study, a diverse mapping panel was used to uncover MTAs associated with seedling vigour and root traits in wheat germinated under moisture deficit stress and control conditions.

### Genome-Wide Association and Candidate Gene Identification

Discovering new genes and genomic regions related to seedling vigour and root traits during the seedling stage can help develop moisture deficit stress-tolerant, high-yielding crop varieties. In this regard, historical wheat germplasm collections stored in gene banks can be a valuable resource for identifying these genes and regions using an association mapping approach. In this study, a diverse panel of 193 wheat genotypes, including advanced breeding lines, commercial cultivars, elite varieties, germplasm core set, and synthetic derivatives was used to map seedling vigour and root traits. Analysis of variance for all the studied traits in both seasons showed significant variation. The trait variations are a prerequisite for genetic studies and a breeding program of the seedling vigour traits.

The discovery of MTAs by GWAS is influenced by the usage of structured populations [50]. Population structure is employed as a covariate in the study to eliminate this impact. Marker-based PCA revealed that there were three subpopulations in the present material. Principal component analysis (PCA) is a widely used method for determining the population structure within a GWAS panel by utilising high-density SNP data [35,51]. With three major clusters and several sub-clusters branching further, the diversity tree based on genetic distance demonstrates that there was substantial diversity. The grouping pattern and the place of origin appear to be related.

All the core set lines are clustered together as one group and the varieties of Indian origin were grouped in another cluster in the GWAS panel [28]. Linkage disequilibrium (LD) decay across genetic distance in a population determines the density of marker coverage required to conduct GWAS. A greater marker density is needed to capture the markers sufficiently close to the causative loci, as shown by a quicker LD decay.

The significant LD block size for the complete genome in the current investigation is 3.49 Mb. The LDs for the subgenomes for the A, B, and D genomes were determined to be 2.48 Mb, 4.29 Mb, and 3.82 Mb, respectively [28]. In agreement with this, Pang et al. [52] reported a huge LD block size of 4.4 Mb. The B genome had the lowest rate of LD decay in the current study. In contrast, the D and A genomes experienced faster LD decay [53], and slower decay in the D genome was reported in earlier studies [27,35,38,52,54]. Population size, selection, mutation, genetic drift, admixtures, non-random mating, recombination frequency, and pollination behaviour are a few variables that might impact LD in various populations, according to Gupta et al. [55] and Vos et al. [56]. The BLINK model under GAPIT, which is superior at finding QTNs and minimising false positives for identifying true associations, was used in a GWAS [57]. At a *p*-value of <0.0001, a total of 235 MTAs were found to be associated with the investigated traits. However, a Bonferroni adjustment was used for stringent selection in order to prevent false positives. A total of 47 stringent markers were identified, including 10 SNPs linked to MGT, AUC, CL, and RD. Under PEG treatment, 27 significant SNPs were found for the variables AUC, SDW, SVI.I, RD, and RV. The multiple comparisons problem is combated by the Bonferroni correction, which minimises type 1 error, or false positives [58].

Only one SNP marker associated with MGT, AX-94471577, was identified to be present near the candidate regions, identified through in silico analysis coding for WD40-repeat-containing domain superfamily, Aspartic peptidase domain superfamily, Protein DCL-like, and UDP-glucuronosyl/UDP-glucosyltransferase. The aspartic peptidase domainsuperfamily plays an important role in the root and stem development of abiotic and biotic stresses [43]. UDP-glucuronosyl/UDP-glucosyltransferase confers drought tolerance in spring wheat [44]. For AUC, AX-95194336, AX-95194973, and AX-94990696 SNPs were reported under the control, whereas AX-94953183 and AX-94639463 were reported in the moisture deficit stress condition in the first year (2020–21). Only one SNP marker, AX-94491917, was associated with AUC in the second year (2021–22) under control conditions. The AX-94953183 marker was located near the *TraesCS6D02G039600* gene which codes for Papain-like cysteine peptidase superfamily. This gene is involved in seed germination, plant growth and development, organ senescence, immunity, and stress response [59]. Among the seedling vigour traits, CL was found to be associated with drought tolerance in wheat due to determining the maximum depth that seeds can be sown, and it is critical for the establishment of the crop [60]. The SNP markers AX-94583923, AX-95017965, and AX-94773224 were associated with CL across the year. Under control conditions, AX-94918971 and AX-94418067 were reported for the CL. AX-94583923 SNP linked to coleoptile situated near the gene (*TraesCS2D02G372500*) on the 2D chromosome is responsible for encoding the Serine carboxypeptidase enzyme and DNA-binding pseudobarrel domain superfamily. These putative genes play a crucial role in enhancing the ability of coleoptiles and roots as well as in the development of different parts of the plant in wheat to tolerate drought conditions [61,62]. The AX-94699286 marker is associated with the gene that codes for the O-methyltransferase domain. This domain increases the content of melatonin, which enhances drought tolerance [63]. The AX-95103885 marker is linked to a gene that encodes a protein belonging to the α/β hydrolase fold and RmlC-like cupin domain superfamily. These candidate genes are responsible for facilitating the mobilisation of lipids during the germination and initial development of seedlings [64,65]. The candidate regions coding for the EF-hand domain, which plays a crucial role in calcium signalling events in plants, particularly in cell division and the normal development of plant root and shoot tips [66], was identified in the vicinity of the marker AX-94394580. Additionally, the MFS transporter superfamily was found to have a role in nitrate signalling and seedling vigour [36] and its coding regions were also identified near the marker AX-94394580. 

Some of the markers associated with SVI.II have revealed a compelling array of crucial genes, emerging as promising candidates for further exploration. Among them, *TraesCS5A02G387800* stands out, encoding the RNA-binding domain superfamily. A wealth of research has highlighted its pivotal role in governing essential processes such as shoot stem cell fates, root growth, gravitropic responses, and embryo development [67]. Given its significance in these vital biological pathways, *TraesCS5A02G387800* emerges as a compelling target for further investigation concerning seedling vigour. The second candidate gene, *TraesCS5A02G387700*, encodes the Helicase superfamily 1/2, ATP-binding domain, and plays a crucial role in regulating plant growth, development, and abiotic stress responses. It achieves this by modulating the degree of membrane lipid peroxidation [68]. Its involvement in such fundamental biological processes underscores its potential relevance to SVI.II, warranting further exploration. The third candidate gene, *TraesCS2D02G474800*, encodes the F-box-like domain superfamily and participates in diverse biological processes, including seed germination and seedling development. Moreover, it plays a specific role in protein degradation through post-translational modification [69]. The significance of this gene in fundamental cellular processes adds to its appeal as a candidate for seedling vigour studies. Another noteworthy gene, *TraesCS2D02G474900*, encodes the APO domain and exhibits higher expression levels in early vigorous wheat cultivars [70]. Given its association with plant vigour and growth, this gene is a compelling candidate for exploring its potential implications in seedling vigour. The candidate genes identified in association with SVI.II hold tremendous promise in illuminating the underlying mechanisms of seedling vigour in wheat. Their potential to provide valuable insights into the regulation of crucial biological processes in wheat makes them compelling targets for further investigation. As standout contenders from the extensive pool of potential candidates, these genes present exciting opportunities to gain a deeper understanding of the intricate mechanisms governing seedling vigour.

SNP marker AX-94446435 was linked with the candidate genes coding for the Pyridoxal phosphate-dependent transferase domain and domain of unknown function DUF1664. The first gene encodes Pyridoxal phosphate-dependent transferase, which is an enzyme that is involved in the biosynthesis of vitamin B6. Vitamin B6 plays a crucial role in plant growth and development. It is involved in various metabolic processes such as amino acid metabolism, hormone biosynthesis, and stress responses [71]. The second gene is involved in the ROS signalling pathway and plays an important role in plant stress response [72,73]. The SNP marker AX-95234949 is located in proximity to the *TraesCS3A02G087200* gene, which codes for the AP2/ERF domain. This domain includes dehydration-responsive element-binding factors (DBFs) that play essential regulatory functions in the plant’s response to abiotic stresses [74] and enhanced drought tolerance and abscisic acid sensitivity during seedling development [75]. Additionally, this marker is associated with several putative candidate genes, namely, NAD(P)-binding domain superfamily, Ribonucleoside hydrolase-like, RAP domain, and F-box-like domain superfamily. The SNP marker AX-94811606 is situated on the 4D chromosome and is associated with candidate genes that encode for P-type ATPase, subfamily IIB. This protein plays a significant role during root epidermis development and early seedling development by participating in sucrose signalling [76].

Olivoto and Nardino [77] proposed a novel method (multi-trait genotype–ideotype distance index: MGIDI) for genotype selection based on information on multiple traits. A total of 193 genotypes were evaluated under the control and moisture deficit condition for seedling vigour and root traits to facilitate the selection of genotypes with high vigour. Accordingly, the MGIDI index identified 29 wheat genotypes from the control condition and 29 genotypes from the moisture deficit condition as the most promising genotypes (Figure 7; Table 3). Additionally, there were genotypes remarkably near the cut-off point, implying their potential for intriguing characteristics. Hence, genotypes in close proximity to this point ought to be given careful consideration [77].

These remarkable findings offer a deeper understanding of the genetic mechanisms governing seedling vigour, root development, plant growth, and stress responses. As researchers continue to explore these candidate genes, their insights hold great potential for future advancements in agriculture and crop breeding strategies, paving the way for more resilient and productive crops in the face of challenging environmental conditions. This work has brought to light the various strategies that wheat employs to cope with drought stress during seed germination and the early stages of growth. The uncovered gene sets have the potential to improve the ability of wheat to withstand drought, which could have positive effects on seedling vigour and root architecture. A significant proportion of the genes discovered in the targeted region are crucial for plant growth and development. Numerous studies of comparable genes in other species have offered insights into the functions linked to these genes. The genes encoding the potential candidate regions have been characterised through extensive annotation, shedding light on their functions within the cell organelles.

A greater comprehension of the genomic landscape and its significance to breeding efforts aimed at developing drought-tolerant varieties of wheat could come through a further investigation that emphasises each of the regions that have been uncovered. Overall, this study opens novel possibilities to strengthen wheat’s resilience to drought stress, which is of the utmost importance for guaranteeing food security and sustainable agriculture on the backdrop of changing climatic circumstances. It also makes significant contributions to the field of plant genetics.

## 4. Materials and Methods

A collection of 193 bread wheat genotypes was used in the current study (Supplementary Table S1). The collection includes elite varieties, commercial cultivars, advanced breeding lines, the core set of germplasm, and synthetic derivatives. Seeds harvested from field experiments conducted during the 2020–2021 and 2021–2022 *Rabi* seasons at the Indian Council of Agricultural Research (ICAR) Institute in New Delhi (28.6550° N latitude, 77.1888° E longitude, elevation 228.61 m) were utilised in our laboratory studies, representing the two distinct seasons. More information about the origins (Supplementary Table S1) and population structure has been published by Devate et al. [28].

### 4.1. Seedling-Vigour-Related Traits and Root Traits Measured for Phenotyping

The germination test was conducted by following the ISTA rules [78]. In a laboratory germination test, a moisture deficit stress condition was created using a polyethylene glycol (PEG 6000) solution. PEG-6000 is a widely recognised and commonly used osmotic agent for inducing drought stress in laboratory experiments. PEG-6000 can create a controlled water deficit in the growth medium, mimicking drought conditions, and helps to investigate the plant’s response to water stress in a controlled environment [79]. Its use provides a consistent and replicable way to study plant drought tolerance mechanisms. Germination tests were conducted with −0.4 MPa concentrations of polyethylene glycol (PEG-6000) with wheat genotypes, as standardised by Vinodkumar [80] following Michel and Kaufmann’s methodology [81]. After being surface sterilised with sodium hypochlorite (NaOCl) at 3% for 10 min, seeds were washed three times with double-distilled water. The germination tests were conducted in Petri dishes (15 cm in diameter) with 2 replications for each genotype under study. A total of 50 seeds of each genotype were put out in each replication on two layers of Whatman no. 1 filter paper. To each Petri dish, ten millilitres of PEG solution were added. However, in the control set, double-distilled water was added in the place of the PEG solution. Both sets of Petri dishes were allowed to germinate for 8 days in a germination chamber at 20 ± 2 °C and 70–80% RH. Initially, 10 mL of PEG solution was added to the double-layered filter paper in a 15 cm Petri plate for the water deficit stress treatment [82]. Subsequently, additional PEG solution was added as needed to maintain the desired water potential, while in the control set, double-distilled water was added to maintain the optimum moisture level. The final count was taken on the 8th day and the number of normal seedlings, i.e., seedlings that had all their essential structures (root system and shots) with potential for continuing their development and giving rise to normal plants, was used to calculate the standard germination percentage. The radicle emergence test was performed following the ISTA rules [78] by using dehusked seeds. The seeds were placed to germinate as in the standard germination test. Radicle emergence was ascertained by measuring the growth of a 2 mm radicle after 48 h at 20 ± 2 °C and 70–80% RH. The mean germination time was calculated by using the formula:

MGT = Σ(n × d)/N

where n is the number of seeds germinated on each day, d is the number of days from the beginning of the test, and N is the total number of seeds germinated at the end of the test [83]. 

The germination curve was produced by fitting the daily radicle/coleoptile emergence (≥2 mm) data using the four-parameter Hill function, as described by El-Kassaby et al. [84], using Germinator_curve-fitting1.0.xls. by Joosen et al. [85], and the area under the fitted curve up to 192 h (8 days) after imbibition was considered as the AUC value. Ten normal seedlings were collected after the germination test’s final count from each replication to measure the coleoptile length (cm) and seedling length (cm). The seedling dry weight (mg^−1^) was estimated after taking the final count of the germination test. Ten normal seedlings from each replication were taken out, washed, and dried overnight at 80 + 1 °C. Seedling vigour indices were determined following Abdul-Baki and Anderson [86], by using the following formulas:

Seedling vigour index−I=Germination % × Seed length (cm)

and

Seedling vigour index−II=Germination % × Seed dry weight (mg)

Seedling vigour index-I calculates seedling vigour by multiplying the germination percentage with seedling length, while seedling vigour index-II incorporates the seedling dry weight, offering a more comprehensive measure that accounts for the seedling’s actual biomass, encompassing both structural and physiological aspects of early seedling growth, thus enhancing the accuracy of the seedling health and vigour assessment.

The roots were gently removed on day 8 by cutting them at the collar area with a sharp blade. The extracted roots were then placed in a tray filled with distilled water for the phenotyping of root traits. The roots of each genotype were then individually scanned in an Epson Perfection V 700 Photo^®^ flatbed scanner at a resolution of 400 dpi modified for this purpose (Regent Instruments Inc., Quebec, QC, Canada) as per the manufacturer’s guidelines. The root images from the scanner were analysed with customised software WinRHIZO™ (Regent Instruments Inc., Quebec, QC, Canada) [87]. Various root trait data were recorded by the software, such as total root length, root surface area, root diameter, root volume, and number of tips and forks.

The multi-trait genotype–ideotype distance index (MGIDI) was used to select the superior genotypes under both the control and moisture deficit conditions based on seedling vigour and root traits by following Olivoto and Nardino [77].

### 4.2. Genotyping

Genomic DNA from leaf samples of all 193 genotypes was isolated using the cetyltrimethylammonium bromide (CTAB) extraction method [88] followed by a DNA quality check through 0.8% agarose gel electrophoresis. Genotyping was carried out using the Axiom Wheat Breeder’s Genotyping Array (Affymetrix, Santa Clara, CA, USA), with 35,143 SNPs, following standard protocols. Allele calling was carried out using the Affymetrix proprietary software package Axiom Analysis Suite, following the Axiom^®^ Best Practices genotyping workflow (https://media.affymetrix.com/support/downloads/manuals/axiom_analysis_suite_user_guide.pdf (Accessed on 24 February 2022)). The SNPs were filtered, and monomorphic markers and markers with a minor allele frequency (MAF) of 5%, missing data of more than 10%, and heterozygote frequency greater than 50% were eliminated from the study. The remaining 13,947 SNPs were analysed further, as used by Devate et al. [28].

### 4.3. Statistical Analyses of Phenotypic Data

The descriptive statistics and frequency distribution were analysed to check the range of variability among the traits. The mean, standard deviation, and range for each trait were calculated and are given in Table 1. Analysis of variance of trait (Y) was calculated across the replication over the season using the ‘agricolae’ package [89] in R with the following models

Y = Variety + Replication + Season + Treatment + Variety × Treatment + Variety ×Season + e

Pearson correlation coefficients and a PCA biplot were also calculated to determine the relationships among the traits between treatments. The Pearson’s correlation coefficient among the studied traits was calculated and graphically represented using an R package ‘corrplot’ [90]. Phenotypic-based PCA was performed using the R package “FactoMineR version 2.4” (multivariate exploratory data analysis and data mining) by Husson et al. [91]. Graphical representation of the PCA results was achieved with the R package “factoextra version 1.0.7” [92]. Graphical representations of the phenotypic data, including the frequency distribution, were created using the “rcompanion” package [93] in the R 4.1.2 software.

### 4.4. Diversity, Linkage Disequilibrium, and Association Analysis

The diversity in the GWAS panel was assessed using marker-based principal component analysis (PCA) and neighbour-joining (NJ) dendrogram analysis, and intrachromosomal linkage disequilibrium (LD) was previously published in Devate et al. [28]. The study used molecular-marker-based PCA to demonstrate that PC1 and PC2 corresponded to 54.56% and 25.03% of the variation, respectively. The population was grouped into three subgroups, and a neighbour-joining dendrogram was drawn based on the distance matrix among the genotypes from the GWAS panel. The dendrogram inferred three main clusters branching into many clusters in the population. To avoid false associations occurring as a result of the population structure, PCA-based population grouping was used as a covariate in the association analysis. The marker pairs’ linkage disequilibrium (LD) was computed as r^2^. Using the r^2^-value versus genetic distance in base pairs (bps), the LD decay plot was produced. The whole genome’s 3.49 Mb big LD block size signifies that SNPs in this region function as inheritance blocks. For subgenomes A, B, and D, the LD block sizes were 2.48 Mb, 4.29 Mb, and 3.82 Mb, respectively.

A total of 13,947 filtered SNPs and the mean value for each treatment was calculated from the two seasons’ data for control (well watered) and moisture deficit conditions (PEG-6000 treatment). An overall mean across the treatment was also calculated. Phenotypic data from all the traits were used to identify associated markers with traits using the ‘BLINK’ (Bayesian-information and linkage-disequilibrium iteratively nested keyway) model [57] under GAPIT v3 in R. The BLINK model has been proven to be more precise in locating QTNs and avoiding false positives in uncovering the true associations. In the model, PCA-based population structure was included as a fixed effect to control for the effect of population structure in the analysis. A Q–Q plot was created to assess the fit of the association model by plotting expected vs. observed −*log10(p)* values. Marker trait associations (MTAs) across seasons and combined for RE, GP, MGT, AUC, CL, SL, SDW, SVI.I, SVI.II, RL, RSA, RD, RV, RT, and RF were found to have significant *p*-values using Bonferroni correction (*p* = 0.05/total number of markers, −log(*p*) = 5.45) to ensure stringent selection of MTAs. 

### 4.5. In Silico and Gene Annotation

Associated markers identified using GWAS were subjected to a basic local alignment search tool (BLAST) search using the sequence information of the markers. The BLAST search was carried out using the data web service Ensembl Plants [94] (https://plants.ensembl.org/Triticum_aestivum/Tools/Blast (Accessed on 25 may 2023)) against the bread wheat reference genome IWGSC (RefSeq v1.0). To identify the candidate genes associated with significant SNPs, gene coding regions located within the 100 kb flanking region of the MTAs were considered. Further gene annotation of identified genes was carried out to know their biological process, cellular components, and molecular functions (Supplementary Table S4).

## 5. Conclusions

The current study examines how early-growth seedling vigour traits and root attributes respond to moisture deficit stress. The relatively significant variations in the observed parameters highlight their potential as criteria for selection for effectively evaluating a large number of genotypes. The complex genetic nature of these traits is shown by the genetic analysis of these traits under moisture deficit stress. We detected genes that are sensitive to moisture deficit stress and that encode a variety of proteins that influence germination, post-germination processes, and plant responses. These genes, either constitutively or adaptively, perform various roles in seed germination under both normal conditions and drought-induced situations. Understanding early drought stress responses in wheat and identifying the genes that are associated with adaptation can help with genetic modification in boosting plant tolerance to stress. These results suggest that throughout their early growth, wheat seedlings deploy sophisticated processes for adaptability. However, additional functional validation is required to understand the genetic control of moisture deficit stress during early developmental stages (germination and seedling) in wheat and to validate the associations and genes identified in this study. Furthermore, testing in the field may prove necessary to assess the agronomic significance of our findings.

## Figures and Tables

**Table 1:** Descriptive statistics, analysis of variance (ANOVA) for seedling-vigour-related traits, and root traits across the two seasons under control and moisture deficit stress condition treatments.

SV	Genotype	Replication	Season	Treatment	Genotype ×Treatment	Genotype × Season	Mean ± SE	CV	LSD@5%
DF	192	1	1	1	192	192			
RE	0.16 ***	0.31 ***	0.05	160.45 ***	0.13 ***	0.12 ***	1.04 ± 0.01	13.48	0.16
GP	0.07 ***	0	0.88 ***	56.01 ***	0.06 ***	0.06 ***	1.25 ± 0.01	7.36	0.12
MGT	0.43 ***	0	4.11 ***	106.38 ***	0.50 ***	0.39 ***	2.33 ± 0.02	9.94	0.45
AUC	1121.25 ***	769.27	20,640.52 ***	725,833.40 ***	762.79 ***	1137.99 ***	128.45 ± 0.85	9.22	15.56
CL	2.03 ***	0	1.87 ***	952.09 ***	0.57 ***	0.71 ***	2.72 ± 0.04	18.53	0.32
SL	60.37 ***	0.18	2891.82 ***	55,116.34 ***	17.78 ***	54.25 ***	17.18 ± 0.20	15.99	1.9
SDW	53.17 ***	2.89	1687.35 ***	6769.01 ***	26.55 ***	46.72 ***	10.93 ± 0.19	23.59	2.33
SVI.I	452,276.98 ***	2686.34	11,069,977.94 ***	746,530,121.25 ***	253,856.34 ***	354,442.38 ***	1525.99 ± 17.12	15.58	231.62
SVI.II	473,529.52 ***	29,882.92	21,176,293.17 ***	138,496,911.33 ***	304,158.34 ***	433,009.55 ***	954.73 ± 17.51	25.48	247.34
RL	116.72 ***	0.06	408.05 ***	355,674.09 ***	126.08 ***	116.82 ***	25.79 ± 0.27	14.81	5.01
RSA	3.02 ***	0.04	0.05	10,467.83 ***	3.12 ***	2.93 ***	4.34 ± 0.04	14.16	0.77
RD	0.24 ***	0	0.09	5.14 ***	0.19 ***	0.25 ***	0.58 ± 0.01	29.62	0.2
RV	0.03 ***	0	0.19 ***	1.25 ***	0.01 ***	0.04 ***	0.08 ± 0.00	75.41	0.04
RT	1039.16 ***	0.3	1256.46 **	250,152.40 ***	832.06 ***	968.04 ***	33.66 ± 0.82	33.86	11.21
RF	1031.77 ***	15.55	465.91 ***	271,075.1	801.44 ***	946.31 ***	31.70 ± 0.82	35.83	11.98

*** 0.1% level of significance; ** 1% level of significance; SV: source of variation; DF: degrees of freedom; Treatment: PEG-6000 treatment, RE: radicle emergence (%); GP: germination percentage (%); MGT: mean germination time (d); AUC: area under the curve; CL: coleoptile length (cm); SL: seedling length (cm); SDW: seedling dry weight (mg); SVI.I: seedling vigour index-I; SVI.II: seedling vigour index-II, RL; total root length (cm); RSA: root surface area (cm^2^); RD: average root diameter mm; RV: root volume (cm^3^); RT: number of root tips; RF: number of root forks; RE and GP are arc sine transformed.

**Table 2:** Significant marker-trait associations (MTAs) with a Bonferroni-corrected *p*-value (–log10(*p*) > 5.45) for traits under study across seasons.

S.No	Trait	MTA	Chromosome	Physical Position (Mb)	–log_10_(*p*)	PVE (%)
1	ALL_CL	AX-94583923	2D	476.70	10.4828	10.83
2	ALL_CL	AX-95017965	2B	19.07	10.1716	24.16
3	ALL_CL	AX-94773224	6B	650.60	7.8627	8.62
4	ALL_SDW	AX-94699286	4A	660.99	7.5173	28.40
5	ALL_SDW	AX-95103885	6D	13.98	5.5648	29.28
6	ALL_RD	AX-94446435	3D	43.02	9.7491	0.00
7	ALL_RD	AX-94470331	4D	5.69	8.4061	89.43
8	ALL_RD	AX-95234949	3A	56.39	8.0834	0.13
9	ALL_RD	AX-94492491	7A	581.85	7.6457	0.27
10	ALL_RD	AX-94811606	4D	53.24	6.4626	0.24
11	C_MGT	AX-94471577	4A	1.89	5.6717	99.87
12	C_CL	AX-94918971	6D	430.40	10.3226	13.51
13	C_CL	AX-94418067	2B	788.24	6.3630	8.64
14	D_SDW	AX-94742835	2D	90.24	9.6563	47.13
15	D_SDW	AX-94394580	5A	700.44	8.9080	20.51
16	D_SDW	AX-94906386	4D	323.58	8.2085	12.76
17	D_SDW	AX-94455747	4B	562.36	7.4682	0.97
18	D_SDW	AX-94510892	6A	112.59	7.1003	0.93
19	D_SDW	AX-94651675	3B	816.60	5.9655	0.40
20	D_SVI-II	AX-94926133	5A	584.71	7.0389	7.72
21	D_SVI-II	AX-94721930	1D	423.92	5.9759	6.53
22	D_RD	AX-95227434	7D	312.98	7.0386	0.22
23	S1C_AUC	AX-95194336	2B	9.62	11.7447	92.40
24	S1C_AUC	AX-95194973	7B	105.01	6.1170	0.90
25	S1C_AUC	AX-94990696	5D	90.00	5.9162	0.39
26	S1D_AUC	AX-94953183	6D	16.30	6.4548	7.94
27	S1D_AUC	AX-94639463	3D	40.01	5.7741	7.25
28	S1D_SDW	AX-94620141	2D	14.77	11.8387	0.28
29	S1D_SDW	AX-94691215	5D	545.93	10.4072	0.07
30	S1D_SDW	AX-95094238	5A	510.89	9.9641	0.01
31	S1D_SDW	AX-94574509	1A	9.58	9.2637	0.17
32	S1D_SDW	AX-94475237	7A	353.21	9.1639	1.16
33	S1D_SDW	AX-94814248	2D	51.26	8.6749	0.28
34	S1D_SDW	AX-94635936	5D	26.79	8.4742	0.48
35	S1D_SDW	AX-95132381	6A	105.01	7.2472	1.20
36	S1D_SDW	AX-95232641	5B	449.13	7.2043	0.11
37	S1D_SDW	AX-94661897	6B	688.30	6.3670	24.15
38	S1D_SDW	AX-94873710	3A	20.00	5.9096	0.08
39	S1D_SVI-II	AX-94485323	2D	577.60	6.2636	0.09
40	S1D_SVI-II	AX-95204453	3D	49.44	5.5436	0.23
41	S2C_AUC	AX-94491917	3D	429.63	7.3757	78.78
42	S2C_CL	AX-94526026	1B	509.13	5.9038	4.98
43	S2C_SL	AX-94515822	1B	638.93	5.7601	79.58
44	S2C_RD	AX-94575638	6A	531.10	6.9227	16.82
45	S2D_RV	AX-95252696	7B	706.85	6.9025	47.14
46	S2D_RV	AX-95133300	4A	68.69	6.8765	8.96
47	S2D_RD	AX-94449793	5B	547.58	7.1299	40.01

All: control and moisture deficit stress conditions combined across the seasons; C: control; D: moisture deficit stress condition; S1: season 1; S2: season 2.

**Table 3:** Genotype selected using MGIDI for seedling vigour and root traits under control and moisture deficit conditions.

S.No.	Control	Moisture Deficit Condition	S.No.	Control	Moisture Deficit Condition
1	CS86	HB208	16	HD3362	**NW1014**
2	Raj4083	RAJ4238	17	PBW681	HD3226
3	HD3354	CS129	18	MP1203	VL907
4	**NW1014**	**CS28**	19	CS23	CS90
5	CS71	HDR77	20	**Frontana**	PBW723
6	DBW39	LOKBOLD	21	CS69	HI1544
7	**HI1634**	HD2987	22	CS92	**HD2932**
8	**HS490**	CS128	23	HD2189	**Frontana**
9	NI5439	**ISTSAMNYT406**	24	HD2985	**HS490**
10	PBW65	CS93	25	CS14	PBW752
11	WH1105	CS145	26	**ISTSAMNYT406**	DBW17
12	HW2004	**HI1634**	27	**CS28**	**RAJ4229**
13	SAFEDLERMA	CS94	28	DBW187	CS78
14	HD3249	HI1612	29	**RAJ4229**	CS42
15	**HD2932**	PBW771			

Note: Varieties identified under both control and stress condition are highlighted with bold letters.

## Data Availability

Phenotypic data used in this study is available in supplementary file as “Phenotypic data” Genotypic data is available at https://doi.org/10.5061/dryad.0cfxpnw6c (accessed on 1 September 2022).

## Supplementary Materials

The following supporting information can be downloaded at: https://www.mdpi.com/article/10.3390/genes14101902/s1, Phenotypic data used for study; Supplementary Table S1: Genotypes used for GWAS along with their pedigree details; Supplementary Table S2. Descriptive statistics, analysis of variance (ANOVA) for seedling vigour-related traits and root traits across the two seasons under control condition; Supplementary Table S3. Descriptive statistics, analysis of variance (ANOVA) for seedling vigour-related traits and root traits across the two seasons under moisture deficit stress condition; Supplementary Table S4. Putative candidate genes and molecular functions in the 100 kb Region of linked SNPs.
