# Supplementary material for: Unravelling the Genetic Basis of Moisture Deficit Stress Tolerance in Wheat for Seedling Vigour-Related Traits and Root Traits Using Genome-Wide Association Study
Source: Genes (Basel). 2023 Sep 30;14(10):1902. doi: 10.3390/genes14101902 (PMC10606372; doi:10.3390/genes14101902)
Supplement: Supplementary file 1 [file genes-14-01902-s001.zip › Supplementary Table 1.pdf]

Supplementary table1: Genotypes used for GWAS along with their pedigree details

Genotypes	Pedigree information	Accession type	Developing Institute	Year of Release
C591	PENJAB-TYPE-8-B/PENJAB-TYPE-9	Indian variety	Department of Agriculture,Punjab	-
CBW38	CNDO/R 143/ENTE /MEXI_2/3/Ae.SQU ARROSA (TAUS)/ 4/WEAVER/5/2*PA STOR	Indian variety	IIWBR, Karnal, Haryana	2009
CG1029	-	Indian variety	-	
Chirya-3	CHINESE-SPRING/AG.CU//GLENNSON-81/3/ALONDRA/PAVON-76/4/NINGMAI-4/OLESEN//ALONDRA/YANGMAI-4	Exotic Variety	Mexico	1992
CS100	PUB94.15.1.12/FRTL	Exotic line	CIMMYT, Mexico	
CS110	NORD-DESPREZ/VG-9144//KALYANSONA/BLUEBIRD/3/YACO/4/VEERY-5	Exotic line	CIMMYT, Mexico	-
CS112	76ECN44/76ECN36//MADDEN/6*RAC177	Exotic line	CIMMYT, Mexico	-
CS128	BAV92/SERI	Exotic line	CIMMYT, Mexico	-
CS129	SB025	Exotic line	CIMMYT, Mexico	-
CS134	PASTOR//HXL7573/2*BAU/3/MEX94.2.19//ATTILA/3*BCN	Exotic line	CIMMYT, Mexico	
CS14	DRYSDALE	Exotic line	CIMMYT, Mexico	
CS141	PUB94.15.1.12/3/FRTL//ATTILA/3*BCN	Exotic line	CIMMYT, Mexico	
CS143	AUS30354	Exotic line	CIMMYT, Mexico	-
CS144	CIANO-79/PARULA//CHILERO	Exotic line	CIMMYT, Mexico	-
CS145	LOVE-HH-129	Exotic line	CIMMYT, Mexico	
CS15	SOKOLL	Exotic line	CIMMYT, Mexico	
CS17	BERKUT	Exotic line	CIMMYT, Mexico	
CS18	KUKRI/EXCALIBUR	Exotic line	CIMMYT, Mexico	
CS19	MEX94.2.19/PUB94.15.1.12	Exotic line	CIMMYT, Mexico	-
CS23	PASTOR//HXL7573/2*BAU/3/CMH82.575/CMH82.801	Exotic line	CIMMYT, Mexico	
CS27	PARUS/3/CHEN/AE.SQ//2*OPATA	Exotic line	CIMMYT, Mexico	
CS28	W15.92/4/PASTOR//HXL7573/2*BAU/3/WBLL1	Exotic line	CIMMYT, Mexico	-
CS32	BAV92/SERI	Exotic line	CIMMYT, Mexico	-
CS38	PFAU/SERI-82(CID-93300//BOBWHITE	Exotic line	CIMMYT, Mexico	-

CS4	JANZ	Exotic line	CIMMYT, Mexico	
CS40	MEX94.27.1.20/3/SOKOLL//ATTILA/3*BCN	Exotic line	CIMMYT, Mexico	
CS41	SSRT16	Exotic line	CIMMYT, Mexico	-
CS42	BOBWHITE/NACOZARI-76//VEERY/3/BLUEJAY/COCORAQUE-75	Exotic line	CIMMYT, Mexico	-
CS46	BAV92/SERI	Exotic line	CIMMYT, Mexico	-
CS53	OAX93.24.35//SOKOLL/WBLL1	Exotic line	CIMMYT, Mexico	
CS58	BAV92/SERI	Exotic line	CIMMYT, Mexico	
CS64	PUB94.15.1.12/FRTL	Exotic line	CIMMYT, Mexico	
CS66	SERI/BAV92//WBLL1	Exotic line	CIMMYT, Mexico	
CS69	KUKRI/RAC875	Exotic line	CIMMYT, Mexico	-
CS7	SSRT09	Exotic line	CIMMYT, Mexico	
CS71	HARTOG	Exotic line	CIMMYT, Mexico	
CS74	AUS30518	Exotic line	CIMMYT, Mexico	
CS78	VJ99	Exotic line	CIMMYT, Mexico	
CS80	SSRT65	Exotic line	CIMMYT, Mexico	
CS86	SB187	Exotic line	CIMMYT, Mexico	-
CS89	RAC 655	Exotic line	CIMMYT, Mexico	
CS90	AUS30523	Exotic line	CIMMYT, Mexico	
CS92	KRICHAUFF	Exotic line	CIMMYT, Mexico	
CS93	PASTOR//HXL7573/2*BAU/3/MEX94.2.19//ATTILA/3*BCN	Exotic line	CIMMYT, Mexico	
CS94	MEX94.2.19//SOKOLL/WBLL1	Exotic line	CIMMYT, Mexico	-
DBW107	-	Indian variety	-	
DBW110	KIRITATI/4/2*SERI1B*2/3/KAUZ*2/BOW//KAUZ	Indian variety	IIWBR, Karnal, Haryana	2015
DBW14	RAJ 3765/PBW 343	Indian variety	IIWBR, Karnal, Haryana	2003
DBW16	RAJ 3765/WR 484//HUW 468	Indian variety	IIWBR, Karnal, Haryana	2006
DBW17	CMH79A.95/3*CNO 79//RAJ3777	Indian variety	IIWBR, Karnal, Haryana	2007
DBW173	KAUZ/AA//KAUZ/P BW602	Indian variety	IIWBR, Karnal, Haryana	2018
DBW187	NAC/TH/AC//3*PVN/3/MIRLO/BUC/4/ 2*PASTOR/5/KACHU/6/KACHU	Indian variety	IIWBR, Karnal, Haryana	2019
DBW222	KACHU/SAUAL*ATTILA*2/PBW65/6/PVN//CAR422/ ANA/5/BOW/CROW//BUC/PVN/3/YR/4/TRAP#1/7/ATTILA/2*PASTOR	Indian variety	IIWBR, Karnal, Haryana	2020

DBW296	-	Indian variety	-	
DBW303	-	Indian variety	-	
DBW39	ATTILA/HUI	Indian variety	IIWBR, Karnal, Haryana	2010
DBW43	BA BAX/LR42// BABAX*2/3/VIVITSI	Breeding line	IIWBR, Karnal, Haryana	-
DBW51	SITELLA/MILAN	Breeding line	IIWBR, Karnal, Haryana	-
DBW88	KAUZ//ALTAR84/AOS/3/MILAN/KAUZ/4/HUITES	Indian variety	IIWBR, Karnal, Haryana	2014
DL1266-2	-	Indian variety	-	
DL153-2	TANORI 71/ NP 890	Indian variety	IARI, New Delhi	1985
DL788-2	K7537/HD2160/ HD2278//L24/K4.14	Indian variety	IARI, New Delhi	1997
Drysdale	HARTOG*3/QUARRION	Exotic Variety	Australia	2001
Excalibur	RAC-177(Sr26)/UNICULM-492//RAC-311-S	Exotic Variety	Australia	2001
Frontana	FRONTEIRA/MENTANA	Exotic Variety	brazil	1930
GW273	CPAN 2084/VW 205	Indian variety	SDAU RS, Vijapur, Gujarath	1998
GW322	GW 173/GW 196	Indian variety	SDAU RS, Vijapur, Gujarath	2002
GW366	DL 802-3/GW 232	Indian variety	JAU, Junagadh, Gujarath	2007
GW451	GW324/4/CROC_1/AE.RA RS,QUARROSA(205)//J U Vijapur P/ JY/3/SKAUZ /4/KAUZ/5/GW339	Indian variety	JAU, Junagadh, Gujarath	2016
GW513	-	Indian variety	-	
Halna	HD 1982/K816	Indian variety	CSAUA&T, Kanpur, Uttar Pradesh	2001
HB208	SPO/MTA//MQ /2*RNW//3/PJ' S'/P14/KT54B	Indian variety	Bhowali, Uttarakhand	1982
HD2009	LR 64A /NAI 60	Indian variety	IARI, New Delhi	1975
HD2189	HD 1963 / HD 1931	Indian variety	IARI, New Delhi	1980
HD2329	HD 1962/E 4870/3/K 65/5/HD1553/4/UP262	Indian variety	IARI, New Delhi	1985
HD2687	CPAN2009/HD 2329	Indian variety	IARI, New Delhi	1999
HD2733	ATTILA /3/TUI /CARC //CHEN / CHTO /4/ATTILA	Indian variety	IARI, New Delhi	2001
HD2824	PTO-1 /CNO79/PRL /GAA /3/HD 1951	Indian variety	IARI, New Delhi	2004
HD2833	PBW 226 / HW 1042//HD 2285	Indian variety	IARI, New Delhi	2006
HD2851	CPAN 3004/WR426//HW 2007	Indian variety	IARI, New Delhi	2005

HD2864	DL 509-2/ DL 377-8	Indian variety	IARI, New Delhi	2005
HD2888	C306/T.sphaerococcum//HW 2004	Indian variety	IARI, New Delhi	2006
HD2894	HW 2002/WR 196	Indian variety	IARI, New Delhi	2008
HD2932	KAUZ/STAR//HD 2643	Indian variety	IARI, New Delhi	2008
HD2967	ALD/COC//URES/HD216 0M/HD2278	Indian variety	IARI, New Delhi	2011
HD2982	-	Breeding line	IARI, New Delhi	-
HD2985	PBW 343/ PASTOR	Indian variety	IARI, New Delhi	2011
HD2987	HI1011/HD2348//M ENDOS//IWP 72/DL153-2	Indian variety	IARI, New Delhi	2011
HD3043	-	Indian variety	IARI, New Delhi	
HD3059	KAUZ//ALTAR84/ AOS/3/MILAN/KA UZ/4/HUITES	Indian variety	IARI, New Delhi	2013
HD3086	DBW14/HD2733//HUW468	Indian variety	IARI, New Delhi	2014
HD3090	SFW/VAISHALI// UP2425	Indian variety	IARI, New Delhi	2014
HD3118	ATTILA*2/PBW65 //WBLL1*2/TUKU RU	Indian variety	IARI, New Delhi	2015
HD3171	PBW 343/HD 2879	Indian variety	IARI, New Delhi	2017
HD3226	GRACKLE/HD2894	Indian variety	IARI, New Delhi	2019
HD3237	HD3016/HD2967	Indian variety	IARI, New Delhi	2019
HD3249	PBW343*2/KUKUNA//SRTU/3/PBW343*2/KHVAKI	Indian variety	IARI, New Delhi	2020
HD3354	QUAIU#1/3/T.DICOCCONPI94625/AE.SQ(372)//3*PASTOR/4/ QUAIU#2/5/VALI/6/B ECARD/QUAIU#1	Breeding line	IARI, New Delhi	-
HD3362	-	Indian variety	IARI, New Delhi	
HDR77	PARTIZANKA /HD 2204 //HD2204	Indian variety	IARI, New Delhi	1990
HI 1500 (AMRITA)	HW 2002*2// STREMPALLI/PN C 5	Indian variety	IARI RS, Indore, Madya Pradesh	2003
HI1500	HW 2002*2// STREMPALLI/PN C 5	Indian variety	IARI RS, Indore, Madya Pradesh	2003
HI1531	HI 1182/ CPAN 1990	Indian variety	IARI RS, Indore, Madya Pradesh	2006
HI1544	HINDI62/BOBWHI TE/CPAN 2099	Indian variety	IARI RS, Indore, Madya Pradesh	2008
HI1563	MACS 2496*2/MC 10	Indian variety	IARI RS, Indore, Madya Pradesh	2011

HI1605	BOW/VEE/5/ND/VG9 144//KAL/BB/3/YACO /4/CHIL/6/ CASKOR/3 /CROC_1/AE.SQUAR ROSA(224)//OPATA/ 7/PASTOR/MLAN/KAUZ/3/BAV92	Indian variety	IARI RS, Indore, Madya Pradesh	2017
HI1612	Kauz//Altar84/AO S/3/Milan/Kauz/4/Huites	Indian variety	IARI RS, Indore, Madya Pradesh	2018
HI1628	-	Indian variety	IARI RS, Indore, Madya Pradesh	
HI1633	-	Indian variety	IARI RS, Indore, Madya Pradesh	
HI1634	-	Indian variety	IARI RS, Indore, Madya Pradesh	
HI617	Selection from C 306	Indian variety	IARI RS, Indore, Madya Pradesh	1982
Hindi-62	PBW-226/HW-1042//HD-2285	Indian variety	Introduction from India to Egypt	-
Hira	YT54/N 10BIISN64	Indian variety	IARI, New Delhi	1972
HS490	HS 364/HPW 114//HS 240/HS 346	Indian variety	IARI RS, Shimla, Himachal Pradesh	2009
HS543	-	Breeding line	IARI RS, Shimla, Himachal Pradesh	-
HS611	69-1776/663//2*BCN/3/7*BCN/4/PARUS/PASTOR	Breeding line	IARI RS, Shimla, Himachal Pradesh	-
HUW468	CPAN-1962 / TONI //LIRA'S'/ PRL'S'	Indian variety	BHU, Varanasi, Uttar Pradesh	1999
HUW510	HD 2278/HUW 234//DL 230-16	Indian variety	BHU, Varanasi, Uttar Pradesh	2001
HUW666	-	Indian variety	BHU, Varanasi, Uttar Pradesh	-
HUW838	-	Indian variety	-	
HW2004	C 306 *7 //TR 380-14 #7 /3 AG14	Indian variety	IARI RS, Wellington, Tamilnadu	1997
HW2045	HD 2402 *6 /SUNSTAR *6 /C-80-1	Indian variety	IARI RS, Wellington, Tamilnadu	2002
IC42	-	Uncharacterized	-	-
IC43	-	Uncharacterized	-	-
IST SAMNYT 406	Exotic Collection	Breeding line	Exotic Collection	-

Janz	3-AG-3/4*CONDOR//COOK	Exotic Variety	Australia	1989
JAUW672	-	Indian variety	-	
K1006	PBW343/HP1731	Indian variety	CSAUA&T, Kanpur, Uttar Pradesh	2014
K1317	K0307/K9162	Indian variety	CSAUA&T, Kanpur, Uttar Pradesh	2018
K65	C591/NP773	Indian variety	CSAUA&T, Kanpur, Uttar Pradesh	1974
K88	VEERY "S"/WL 711	Indian variety	CSAUA&T, Kanpur, Uttar Pradesh	1993
K9465	-	Indian variety	-	
Kachu #1	KAUZ//ALTAR-84/(AOS)AWNED-ONAS/3/MILAN/KAUZ/4/HUITES	Exotic Variety	CIMMYT, Mexico	2012
Kharchia65	KHARCHIA LOCAL/EG 953	Indian variety	RARI, Durgapura, Rajasthan	1970
Kukri	76ECN44/76ECN36//MADDEN/6*RAC177	Exotic Variety	Australia	1999
Kundan	TANORI 71/ NP 890	Indian variety	IARI, New Delhi	1985
Lal Bahadur	S 54723 * RS 31-1 ML 293 BB*KAL2 ML 319 CNO-KAL*CD1 (KALINIA* INIA-BB) ML328 BB-KAL2 ML 408 RONCHA* KAL-NOR67 ML 414 TOB-INIA*KAL	Indian variety	RARI, Durgapura, Rajasthan	1971
Lerma Rojo	Y50/N 10B//L 52/3/2*LR	Indian variety	IARI, New Delhi	1969
Lok Bold	S308/S311	Indian variety	Lok Bharti, Sanosara, Gujarath	-
Lok1	S308/S331	Indian variety	Lok Bharti, Sanosara, Gujarath	1982
Lok67	-	Indian variety	-	-
MACS6222	-	Indian variety	-	
MP1203	FASN/2 TEPOKA/3/ CHEN/A.SQUARRO SA/TA	Indian variety	JNKVV RS, Powarkheda, Madya Pradesh	2009
MP1358	-	Indian variety	-	
MP3224	MP-403-2/GW-322	Breeding line	JNKVV RS, Powarkheda, Madya Pradesh	-
NI5439	REMP 80/3*NP 710	Indian variety	MPKV RS, Niphad, Madya pradesh	1975
NIAW3170	-	Indian variety	-	

NIAW34	CNO 79/PRL "S"	Indian variety	MPKV RS, Niphad, Madya pradesh	1997
NP 770	KONOSU/NP-4	Indian variety	IARI, New Delhi	-
NP715	-	Indian variety	-	
NP852	KF/2*NP 761	Indian variety	IARI RS, Pusa, Bihar	1969
NP866	-	Indian variety	-	
NW1014	HAHN 'S'	Indian variety	NDUA&T, Faizabad, Uttar Pradesh	1997
PBW175	HD 2160 /WG 1025	Indian variety	PAU, Ludhiana, Punjab	1989
PBW644	-	Indian variety	-	
PBW65	USA 255/K 816 /3/ WL 202	Indian variety	PAU, Ludhiana, Punjab	1987
PBW681	UP2338/KALYANSONA	Breeding line	PAU, Ludhiana, Punjab	-
PBW689	PBW442/WH576//DWR232	Breeding line	PAU, Ludhiana, Punjab	-
PBW723	PBW343+Lr57/Yr40+Lr37/Yr17	Indian variety	PAU, Ludhiana, Punjab	2017
PBW752	BW621/4/PBW343//. YR10/6*AVOCET/3/3*PBW343/5/PBW621	Indian variety	PAU, Ludhiana, Punjab	2018
PBW771	PBW550//YR15/6*AVOCET/3/2*PBW550)	Indian variety	PAU, Ludhiana, Punjab	2020
Pissilocal	LV-MADHYA-PRADESH	Landrace	IARI RS, Indore, Madya Pradesh	-
RAC875	RAC-655/3/Sr21/4*LANCE//4*BAYONET	Exotic Variety	Australia	-
Raj1482	NAPO-TOB 'S'/8156/KAL-BB	Indian variety	RARI, Durgapura, Rajasthan	1983
Raj3077	HD 2267/RAJ 1482/5/BB/INIA66' S'/NAPO	Indian variety	RARI, Durgapura, Rajasthan	1989
RAJ3765	HD 2402/VL639	Indian variety	RARI, Durgapura, Rajasthan	1996
RAJ4037	DL 788-2/RAJ 3717	Indian variety	RARI, Durgapura, Rajasthan	2004
RAJ4083	-	Indian variety	-	
Raj4120	PBW 343/V 1	Indian variety	RARI, Durgapura, Rajasthan	2009
RAJ4229	HW2048/RAJ4000	Indian variety	RARI, Durgapura, Rajasthan	2012
RAJ4238	HW 2021/RAJ 3765	Indian variety	RARI, Durgapura, Rajasthan	2016

Safed Lerma	Y50//N10B/3/LR 52/3*LR	Indian variety	IARI, New Delhi	1969
Sharbati Sonara	Amber Mutant of S64	Indian variety	IARI, New Delhi	1969
Sunco	SUN-9-E-27*4/3-AG-14//WW-15/3/3*COOK	Exotic Variety	Australia	1986
UP1109	UP 262 /UP 368	Indian variety	GBPUA&T, Pantnagar, Uttarkhand	1987
UP2425	HD 2320/UP 2263	Indian variety	GBPUA&T, Pantnagar, Uttarkhand	1999
UP262	S 308/BAJIO 66	Indian variety	GBPUA&T, Pantnagar, Uttarkhand	1978
VL829	IBWSN149/CPAN 2099	Indian variety	VPKAS, almora, Uttarkhand	2003
VL892	-	Indian variety	-	
VL907	DYBR 1982- 83/842 ABVD 50/VW9365//P BW 343	Indian variety	VPKAS, almora, Uttarkhand	-
Westonia	CO-1190-203/84-W-127-501	Exotic Variety	Australia	1997
WH 730	cpan2092/improved Lok1	Indian variety		
WH1021	NYOT95/SONAK	Indian variety		2007
WH1080	21ST SAWSN151	Indian variety	CCS HAU, Hisar, Haryana	2011
WH1105	-	Indian variety	-	
WH1124	MUNIA/CHTO/AM SEL	Indian variety	CCS HAU, Hisar, Haryana	2014
WH1129	CS/TH.CS//3*PVN/3/MIRLO/BUC/4/MILAN/5/TILHI	Breeding line	CCS HAU, Hisar, Haryana	-
WH1142	-	Indian variety	-	
WH1252	-	Indian variety	-	
WH1270	-	Indian variety	-	
WH5420	-	Indian variety	-	
WR544	KALYANSONA/ HD 1999//HD2204/DW 38	Indian variety	IARI, New Delhi	2005
