# Supplementary material for: Unravelling the Genetic Basis of Moisture Deficit Stress Tolerance in Wheat for Seedling Vigour-Related Traits and Root Traits Using Genome-Wide Association Study
Source: Genes (Basel). 2023 Sep 30;14(10):1902. doi: 10.3390/genes14101902 (PMC10606372; doi:10.3390/genes14101902)
Supplement: Supplementary file 1 [file genes-14-01902-s001.zip › Supplementary table 2 and 3.pdf]

Supplementary Table 2. Descriptive statistics, analysis of variance (ANOVA) for seedling vigour-related traits and root traits across the two seasons under control condition.

SV	Genotype	Replication	Season	Genotype x Season	Mean ± SE	CV	LSD
DF	192	1	1	192			
RE	0.05***	0.55***	1.18***	0.04***	1.36 ± 0.01	7.21	0.14
GP	0.04***	0	0.09**	0.04***	1.44 ± 0.01	7.22	0.14
MGT	0.29	0.18	0.5	0.31	2.06 ± 0.02	25.99	0.75
AUC	277.9***	6.49	181.73	291.42***	150.13 ± 0.6	7.85	16.38
CL	1.75***	0.02	4.23***	0.44***	3.51 ± 0.05	4.33	0.21
SL	53***	0	1011.42***	43.72***	23.16 ± 0.26	4.8	1.54
SDW	26.86***	9.48***	1706.63***	19.21***	13.02 ± 0.19	3.83	0.69
SVI-I	472895.61	2774.56	7000365.84	369197.5	2221.33 ± 24.75	7	216.29
SVI-II	267582.76***	108533.44***	17801520.54***	197680.63***	1254.23 ± 18.62	6.53	113.89
RL	132.12***	0.08	1069.89***	104.17***	40.97 ± 0.41	4.55	2.59
RSA	3.22***	0.01	1.22***	2.65***	6.95 ± 0.06	4.42	0.43
RD	0.03***	0	0*	0.03***	0.53 ± 0.01	4.52	0.03
RV	0.01***	0	0.27***	0.01***	0.11± 0.00	5.65	0.01
RT	993.58***	4.62	485.24***	797.76***	46.39 ±1.13	5	3.23
RF	1124.67***	24.44*	465.26***	897.44***	44.95 ± 1.21	4.95	3.1

***0.1% level of significance; **1% level of significance; *5% level of significance; SV: source of variation; DF: degrees of freedom; Treatment: PEG-6000 treatment, RE: radicle emergence (%); GP: germination percentage (%); MGT: mean germination time (d); AUC: area under the curve; CL: coleoptile length (cm); SL: seedling length (cm); SDW: seedling dry weight (mg); SVI.I: seedling vigour index-I; SVI.II: seedling vigour index-II, RL; total root length (cm); RSA: root surface area (cm^2); RD: average root diameter mm; RV: root volume (cm^3) ; RT: number of root tips; RF: number of root forks; RE and GP were arc sine transformed.

Supplementary Table 3. Descriptive statistics, analysis of variance (ANOVA) for seedling vigour-related traits and root traits across the two seasons under moisture deficit stress condition.

SV	Genotype	Replication	Season	Genotype x Season	Mean ± SE	CV	LSD
DF	192	1	1	192			
RE	0.24***	0.00	1.97***	0.18***	0.72 ± 0.02	10.07	0.10
GP	0.09***	0.00	1.06***	0.07***	1.06 ± 0.01	5.79	0.09
MGT	0.64***	0.20	4.67***	0.47***	2.59 ± 0.03	8.81	0.32
AUC	1606.14***	1345.24***	46940.72***	1477.94***	106.77 ± 1.44	9.64	14.30
CL	0.86***	0.00	15.93***	0.63***	1.94 ± 0.03	4.66	0.13
SL	25.15***	0.29	1957.84***	25.70***	11.21 ± 0.18	5.22	0.81
SDW	52.86***	0.45	281.59***	53.18***	8.83 ± 0.26	5.76	0.71
SVI-I	233237.70***	15869.11	4241503.20***	205689.82***	830.64 ± 17.38	8.42	97.28
SVI-II	510105.11***	7220.51	5238155.25***	522235.83***	655.23 ± 25.71	9.94	90.56
RL	110.67***	0.00	17.15***	132.69***	10.61 ± 0.38	5.07	0.75
RSA	2.93***	0.03*	0.61***	3.19***	1.74 ± 0.06	5.33	0.13
RD	0.41***	0.00	0.23***	0.43***	0.64 ± 0.02	5.45	0.05
RV	0.03***	0.00	0.01***	0.03***	0.05 ± 0.01	9.32	0.01
RT	877.64***	1.89	789.66***	812.67***	20.93 ± 1.07	5.35	1.56
RF	708.54***	0.40	80.2***	784.47***	18.45 ± 0.96	5.55	1.42

***0.1% level of significance; **1% level of significance; *5% level of significance; SV: source of variation; DF: degrees of freedom; Treatment: PEG-6000 treatment, RE: radicle emergence (%); GP: germination percentage (%); MGT: mean germination time (d); AUC: area under the curve; CL: coleoptile length (cm); SL: seedling length (cm); SDW: seedling dry weight (mg); SVI.I: seedling vigour index-I; SVI.II: seedling vigour index-II, RL; total root length (cm); RSA: root surface area (cm^2); RD: average root diameter mm; RV: root volume (cm^3) ; RT: number of root tips; RF: number of root forks; RE and GP were arc sine transformed.
