# Supplementary material for: Unravelling the Genetic Basis of Moisture Deficit Stress Tolerance in Wheat for Seedling Vigour-Related Traits and Root Traits Using Genome-Wide Association Study
Source: Genes (Basel). 2023 Sep 30;14(10):1902. doi: 10.3390/genes14101902 (PMC10606372; doi:10.3390/genes14101902)
Supplement: Supplementary file 1 [file genes-14-01902-s001.zip › Supplementary table 4.pdf]

Supplementary table 4. Putative candidate genes and molecular functions in the 100 kb Region of linked SNPs

Trait	SNP	Chromosome	Position	Transcript ID	Protein	Function
ALL_CL	AX-94583923	2D	476696204	*TraesCS2D02G372500*	Peptidase S10, serine carboxypeptidase	Serine carboxypeptidase plays an important role in the drought tolerance of coleoptiles and the roots of spring wheat [1]
				TraesCS2D02G372700	DNA-binding pseudobarrel domain superfamily	The B3 DNA-binding superfamily plays a significant role in the response to stresses in plants, as well as in the development of different parts of the plant [2]
ALL_CL	AX-95017965	2B	19070631	*TraesCS2B02G042000*	UDP-glucuronosyl/UDP-glucosyltransferase	UDP-glucuronosyl/UDP-glucosyltransferase confer drought tolerance in spring wheat [3]
ALL_CL	AX-94773224	6B	650596466	*TraesCS6B02G375700*	Zinc finger, SIAH-type	Up-regulated gene induced by drought stress in Tibetan hulless barley [4]
ALL_SDW	AX-94699286	4A	660988520	*TraesCS4A02G383100*	O-methyltransferase domain	Overexpression of the wheat *TaCOMT* gene enhances drought tolerance and increases the content of melatonin in transgenic *Arabidopsis* [5]
ALL_SDW	AX-95103885	6D	13975625	*TraesCS6D02G032900*	Alpha/Beta hydrolase fold	Plays a critical role in mediating lipid mobilization during seed germination and growth [6]
				TraesCS6D02G033100	RmlC-like cupin domain superfamily	The *Arabidopsis* cupin domain protein AtPirin1 interacts with the G protein alpha-subunit GPA1 and regulates seed germination and early seedling development [7]
ALL_RD	AX-94446435	3D	43020473	*TraesCS3D02G085400*	Pyridoxal phosphate-dependent transferase	Up-regulated by drought stress in maize [8]
ALL_RD	AX-94470331	4D	5694858	*TraesCS4D02G012200*	26S proteasome non-ATPase regulatory subunit Rpn12	RPN12a is part of the *Arabidopsis* 26S proteasome that controls the stability of one or more of the factors involved in cytokinin regulation [9]

ALL_RD	AX-95234949	3A	56390763	*TraesCS3A02G087200*	AP2/ERF domain	Dehydration responsive element-binding factors (DBFs) belong to the AP2/ERF superfamily and play vital regulatory roles in abiotic stress responses in plants [10]
ALL_RD	AX-94811606	4D	53243459	*TraesCS4D02G079200*	P-type ATPase, subfamily IIB	Role during root epidermis development and early seedling development via sucrose signalling [11]
C_MGT	AX-94471577	4A	1890138	*TraesCS4A02G002000*	WD40-repeat-containing domain superfamily	-
				TraesCS4A02G002200	Protein DCL-like	-
D_SDW	AX-94742835	2D	90243264	*TraesCS2D02G148700*	Transcription elongation factor, TFIIS/CRSP70, N-terminal, sub-type	Transcription factors (TFs) are involved in drought stress tolerance and response via an ABA signalling pathway [12]
D_SDW	AX-94394580	5A	700443596	*TraesCS5A02G545500*	EF-hand domain	Plays a principal role in calcium signalling events in plants which is an essential nutrient for the normal development of the plant root and shoot tips and more specifically in cell division [13]
				TraesCS5A02G545800	Invertase/pectin methylesterase inhibitor domain superfamily	Early development of wheat grain endosperm and outer Layers [14]
				TraesCS5A02G546200	MFS transporter superfamily	Role in nitrate signalling and seedling vigour [15]
D_SVI-II	AX-94926133	5A	584713681	*TraesCS5A02G387800*	RNA-binding domain superfamily	Function in the regulation of shoot stem cell fates, root growth, gravitropic responses and embryo development [16]
				TraesCS5A02G387700	Helicase superfamily 1/2, ATP-binding domain	play crucial roles in plant growth, development, and abiotic stress responses by regulating the degree of membrane lipid peroxidation [17]
S1C_AUC	AX-95194336	2B	9620943	*TraesCS2B02G020200*	P-loop containing nucleoside triphosphate hydrolase	-

S1C_AUC	AX-95194973	7B	105011342	*TraesCS7B02G090900*	Aspartic peptidase domain superfamily	Role in gluten breakdown [18]
S1D_AUC	AX-94953183	6D	16295427	*TraesCS6D02G039600*	Papain-like cysteine peptidase superfamily	Play a role in seed germination, plant growth and development, organ senescence, immunity, and stress response [19]
				TraesCS6D02G039700	Leucine-rich repeat domain superfamily	The leucine-rich repeat receptor like kinases (LRRK) constitute the largest subfamily of receptor-like kinases (RLK), which play critical roles in plant development and stress responses [20]
S1D_SDW	AX-94620141	2D	14772793	*TraesCS2D02G040700*	Protein kinase-like domain superfamily	Role in various plant processes, including growth, development, and stress response [21]
S1D_SDW	AX-95094238	5A	510891163	*TraesCS5A02G302000*	AAA+ ATPase domain	Abiotic stress tolerance [22]
S1D_SDW	AX-94574509	1A	9580120	*TraesCS1A02G018800*	The domain of unknown function DUF4220	DUF genes are associated with seedling development, root development, plant cell growth, and chloroplast movement [23]
				TraesCS1A02G018900	Zinc finger C2H2 superfamily	Key regulator of drought tolerance [24]
				TraesCS1A02G019000	V-ATPase proteolipid subunit C, eukaryotic	Role in root growth [25]
S1D_SDW	AX-95232641	5B	449131902	*TraesCS5B02G265100*	C2 domain superfamily	Upregulated during drought and osmotic stress [26]
S1D_SDW	AX-94873710	3A	20004183	*TraesCS3A02G036400*	ABC transporter-like, ATP-binding domain	It is essential for both root growth and shoot growth in plants [27]
S1D_SVI-II	AX-94485323	2D	577599376	*TraesCS2D02G474800*	F-box-like domain superfamily	The F-box-like domain superfamily plays specific roles in protein degradation by post-translational modification in several biological processes including seed germination and seedling development [28]

				TraesCS2D02G474900	APO domain	APO expressed greater in early vigorous wheat cultivars [29]
S2C_AUC	AX-94491917	3D	429632555	*TraesCS3D02G316300*	LysM domain superfamily	LysM domain-containing proteins modulate stress response and signalling in wheat [30]
S2C_SL	AX-94515822	1B	638930477	*TraesCS1B02G413800*	Bile acid: sodium symporter/arsenical resistance protein Acr3	BILE ACID: SODIUM SYMPORTER FAMILY PROTEIN 2 (BASS2) repress ABI4 expression during seedling development by binding to its promoter [31]

Note; ALL- control and Moisture deficit stress condition across the season, Combined, C: Control, D: Moisture deficit stress condition (PEG-6000 treatment), S1-Season 1, S2- Season 2; AUC: area under the curve; CL: coleoptile length (cm); SL: seedling length (cm); SDW: seedling dry weight (mg); SVI.I: seedling vigour index-I; SVI.II: seedling vigour index-II, RD: average root diameter (mm)

References

1. Miazek, A.; Nykiel, M.; Rybka, K. Drought tolerance depends on the age of the spring wheat seedlings and differentiates patterns of proteinases. *Russ. J. Plant Physiol.* **2017**, *64*, 333-340.

2. Kandeel, M.; Morsy, M.A.; Abd El-Lateef, H.M.; Marzok, M.; El-Beltagi, H.S.; Al Khodair, K.M.; Venugopala, K.N. Genome-Wide Identification of B3 DNA-Binding Superfamily Members (ABI, HIS, ARF, RVL, REM) and Their Involvement in Stress Responses and Development in *Camelina sativa. Agron.* **2023**, *13*(3), 648.

3. Zahra, S.; Hussain, M.; Zulfiqar, S.; Ishfaq, S.; Shaheen, T.; Akhtar, M. EMS-based mutants are useful for enhancing drought tolerance in spring wheat. *Cereal Res. Commun.* **2021**, 1-12.

4. Yuan, S.; Xu, B.; Zhang, J.; Xie, Z.; Cheng, Q.; Yang, Z.; Cai, Q.; Huang, B. Comprehensive analysis of CCCH-type zinc finger family genes facilitates functional gene discovery and reflects recent allopolyploidization event in tetraploid switchgrass. *Genomics* **2015**, *16*, 1-16.

5. Yang, W.J.; Du, Y.T.; Zhou, Y.B.; Chen, J.; Xu, Z.S.; Ma, Y.Z.; Min, D.H. Overexpression of TaCOMT improves melatonin production and enhances drought tolerance in transgenic *Arabidopsis. Int. J. Mol. Sci.* **2019**, *20*(3), 652.

6. Mindrebo, J.T.; Nartey, C.M.; Seto, Y.; Burkart, M.D.; Noel, J.P. Unveiling the functional diversity of the alpha/beta hydrolase superfamily in the plant kingdom. *Curr. Opin. Struct. Biol.* **2016**, *41*, 233-246.

7. Lapik, Y.R.; Kaufman, L.S. The *Arabidopsis* cupin domain protein AtPirin1 interacts with the G protein α-subunit GPA1 and regulates seed germination and early seedling development. *Plant Cell* **2003**, *15*(7), 1578-1590.

8. Khan, H.; Krishnappa, G.; Kumar, S.; Mishra, C.N.; Krishna, H.; Devate, N.B.; Singh, G. P. Genome-wide association study for grain yield and component traits in bread wheat (Triticum aestivum L.). *Front. genet.* **2022**, *13*, 982589.

9. Smalle, J.; Kurepa, J.; Yang, P.; Babiychuk, E.; Kushnir, S.; Durski, A.; Vierstra, R.D. Cytokinin growth responses in *Arabidopsis* involve the 26S proteasome subunit RPN12. *Plant Cell* **2002**, *14*(1), 17-32.

10. Xu, Z.S.; Ni, Z.Y.; Liu, L.; Nie, L.N.; Li, L.C.; Chen, M.; Ma, Y.Z. Characterization of the TaAIDFa gene encoding a CRT/DRE-binding factor responsive to drought, high-salt, and cold stress in wheat. *Mol. Genet. Genom.* **2008**, *280*, 497-508.

11. Garcia Bossi, J.; Kumar, K.; Barberini, M.L.; Domínguez, G.D.; Rondon Guerrero, Y.D.C.; Marino-Busljе, C.; Obertello, M.; Muschietti, J.P.; Estevez, J. M. The role of P-type IIA and P-type IIB Ca2+-ATPases in plant development and growth. *J. Exp. Bot.* **2020**, *71*(4), 1239-1248.

12. Barbosa Amorim, L.L.; Bezerra-Neto, J.P.; da Fonseca do Santos, R.; Ferreira Neto, J.R.C.; Kido, E.A.; Matos, M.; Benko-Iseppon, A.M. Transcription factors involved in plant drought tolerance regulation. *Drought Stress Tolerance in Plants, Vol 2: Molecular and Genetic Perspectives* **2016**, *2*, 315-358.

13. Mohanta, T.K.; Yadav, D.; Khan, A.L.; Hashem, A.; Abd_Allah, E. F.; Al-Harrasi, A. Molecular players of EF-hand containing calcium signaling event in plants. *Int. J. Mol. Sci.* **2019**, *20*(6), 1476.

14. Mehdi, C.; Virginie, L.; Audrey, G.; Axelle, B.; Colette, L.; Helene, R.; Elisabeth, J.; Fabienne, G.; Mathilde, F.A. Cell wall proteome of wheat grain endosperm and outer layers at two key stages of early development. *Int. J. Mol. Sci.* **2019**, *21*(1), 239.

15. Li, W.; He, X.; Chen, Y.; Jing, Y.; Shen, C.; Yang, J.; Teng, W.; Zhao, X.; Hu, W.; Hu, M.; Li, H.; Tong, Y. A wheat transcription factor positively sets seed vigour by regulating the grain nitrate signal. *New Phytol.* **2020,** *225*(4), 1667-1680.

16. Sun, J.; Bie, X.M.; Wang, N.; Zhang, X.S.; Gao, X.Q. Genome-wide identification and expression analysis of YTH domain-containing RNA-binding protein family in common wheat. *BMC Plant Biol*, **2020**, *20*(1), 1-14.

17. Ru, J.N.; Hou, Z.H.; Zheng, L.; Zhao, Q.; Wang, F.Z.; Chen, J.; Xu, Z.S. Genome-wide analysis of DEAD-box RNA helicase family in wheat (*Triticum aestivum*) and functional identification of TaDEAD-box57 in abiotic stress responses. *Front. Plant Sci.*, **2021**, *12*, 797276.

18. Bleukx, W.; Delcour, J.A. A second aspartic proteinase associated with wheat gluten. *J.Cereal Sci.* **2000**, *32*(1), 31-42.

19. Zou, Z.; Xie, G.; Yang, L. Papain-like cysteine protease encoding genes in rubber (*Hevea brasiliensis*): comparative genomics, phylogenetic, and transcriptional profiling analysis. *Planta*, **2017**, *246*, 999-1018.

20. Shumayla, Sharma, S.; Pandey, A.K.; Singh, K.; Upadhyay, S.K. Molecular characterization and global expression analysis of lectin receptor kinases in bread wheat (*Triticum aestivum*). *PLoS One* **2016**, *11*(4), e0153925.

21. Yan, J.; Su, P.; Wei, Z.; Nevo, E.; Kong, L. Genome-wide identification, classification, evolutionary analysis and gene expression patterns of the protein kinase gene family in wheat and *Aegilops tauschii. Lant Mol. Biol*. **2017**, *95*, 227-242.

22. Liu, S.; Cao, X.; Liao, Y.; Chen, R.; Xu, Z.; Gao, X.; Zhu, J. Identification and characterization of a novel abiotic stress responsive ATPase gene from rice. *Plant Omics* **2015**, *8*(2), 169-177.

23. Lv, P.; Wan, J.; Zhang, C.; Hina, A.; Al Amin, G. M.; Begum, N.; Zhao, T. Unraveling the Diverse Roles of Neglected Genes Containing Domains of Unknown Function (DUFs): Progress and Perspective. *Int. J. Mol. Sci.* **2023**, *24*(4), 4187.

24. Cheuk, A.; Ouellet, F.; Houde, M. The barley stripe mosaic virus expression system reveals the wheat C2H2 zinc finger protein TaZFP1B as a key regulator of drought tolerance. *BMC Plant Biol*, **2020**, *20*(1), 1-34.

25. Padmanaban, S.; Lin, X.; Perera, I.; Kawamura, Y.; Sze, H. Differential expression of vacuolar H+-ATPase subunit c genes in tissues active in membrane trafficking and their roles in plant growth as revealed by RNAi. *Plant Physiolo* **2004**, *134*(4), 1514-1526.

26. Wang, J.; Ding, B.; Guo, Y.; Li, M.; Chen, S.; Huang, G.; Xie, X. Overexpression of a wheat phospholipase D gene, TaPLDα, enhances tolerance to drought and osmotic stress in *Arabidopsis* thaliana. *Planta* **2014**, *240*, 103-115.

27. Larsen, P.B.; Cancel, J.; Rounds, M.; Ochoa, V. *Arabidopsis* ALS1 encodes a root tip and stele localized half type ABC transporter required for root growth in an aluminum toxic environment. *Planta* **2007**, *225*, 1447-1458.

28. Hong, M.J.; Kim, J. B.; Seo, Y.W.; Kim, D.Y. F-box genes in the wheat genome and expression profiling in wheat at different developmental stages. *Genes* **2020**, *11*(10), 1154.

29. Hussain, M.I.; Araniti, F.; Schulz, M.; Baerson, S.; Vieites-Álvarez, Y.; Rempelos, L.; and Sánchez-Moreiras, A.M. Benzoxazinoids in wheat allelopathy–From discovery to application for sustainable weed management. *Environ. Exp. Bot.* 2022, *202*, 104997.

30. Singh, K.; Upadhyay, S.K. LysM domain-containing proteins modulate stress response and signalling in *Triticum aestivum* L. *Environ. Exp. Bot.* **2021** *189*, 104558.

31. Zhao, Y.; Ai, X.; Wang, M.; Xiao, L.; Xia, G. A putative pyruvate transporter TaBASS2 positively regulates salinity tolerance in wheat via modulation of ABI4 expression. *BMC Plant Biol* **2016**, *16*, 1-12.
